# A Novel Approach Integrating Hierarchical Clustering and Weighted Combination for Association Study of Multiple Phenotypes and a Genetic Variant

**DOI:** 10.3389/fgene.2021.654804

**Published:** 2021-06-17

**Authors:** Liwan Fu, Yuquan Wang, Tingting Li, Yue-Qing Hu

**Affiliations:** ^1^State Key Laboratory of Genetic Engineering, Human Phenome Institute, Institute of Biostatistics, School of Life Sciences, Fudan University, Shanghai, China; ^2^Center for Non-communicable Disease Management, Beijing Children’s Hospital, Capital Medical University, National Center for Children’s Health, Beijing, China; ^3^Shanghai Center for Mathematical Sciences, Fudan University, Shanghai, China

**Keywords:** GWAS, hierarchical cluster, multiple phenotypes, score test, obesity

## Abstract

As a pivotal research tool, genome-wide association study has successfully identified numerous genetic variants underlying distinct diseases. However, these identified genetic variants only explain a small proportion of the phenotypic variation for certain diseases, suggesting that there are still more genetic signals to be detected. One of the reasons may be that one-phenotype one-variant association study is not so efficient in detecting variants of weak effects. Nowadays, it is increasingly worth noting that joint analysis of multiple phenotypes may boost the statistical power to detect pathogenic variants with weak genetic effects on complex diseases, providing more clues for their underlying biology mechanisms. So a Weighted Combination of multiple phenotypes following Hierarchical Clustering method (WCHC) is proposed for simultaneously analyzing multiple phenotypes in association studies. A series of simulations are conducted, and the results show that WCHC is either the most powerful method or comparable with the most powerful competitor in most of the simulation scenarios. Additionally, we evaluated the performance of WCHC in its application to the obesity-related phenotypes from Atherosclerosis Risk in Communities, and several associated variants are reported.

## Introduction

Traditionally, Genome-Wide Association Studies studies (GWASs) aim to identify genetic variants associated with certain phenotypes for explaining complex diseases (O’Reilly et al., 2012; Yang and Wang, 2012). In GWASs, multiple related phenotypes of diseases are typically collected for getting better understand complex diseases (Yang Q. et al., 2010). For example, hypertension is directly dependent on the magnitudes of Systolic Blood Pressures (SBP) and Diastolic Blood Pressures (DBP) (Yang and Wang, 2012). The correlation coefficient between SBP and DBP is greater than 0.5 in 95% of patients (Gavish et al., 2008), and researchers could acquire SBP and DBP together for studying hypertension. Similarly, Type 2 Diabetes (T2D) study often gathers relevant risk factors and a number of diabetes-related quantitative phenotypes. Moreover, metabolic syndrome encompasses insulin resistance, obesity, atherosclerotic dyslipidemia, and hypertension; and these factors are interrelated to share potential genetic mediators, pathways, and mechanisms (Huang, 2009). In statistical genetics, jointly analyzing multiple phenotypes can enhance the power of association tests to identify genetic markers associated with one or more phenotypes (Aschard et al., 2014). One of the common approaches for analyzing multiple related phenotypes is to conduct single-phenotype separately and report the results for each phenotype (O’Reilly et al., 2012). However, analysis for one phenotype at a time will be inevitably subject to multiple testing corrections, which leads to a power loss in GWAS (Yang Q. et al., 2010). In recent years, joint analysis of multiple phenotypes has become catching on because of its enhanced statistical power in the detection of genetic variants compared to analysis for each phenotype separately (Yang Q. et al., 2010; Aschard et al., 2014).

Nowadays, jointly analyzing multiple phenotypes has been developed rapidly, which is of two categories: univariate analysis and multivariate analysis. Univariate analysis, as the name suggests, combines various test statistics or *p*-values of univariate association analysis by means of different strategies. Recently, some approaches of univariate analysis have been proposed for exploring the association between multiple phenotypes and a genetic variant (van der Sluis et al., 2013; Liang et al., 2016; Yang et al., 2016). For example, Kwak et al. (Kwak and Pan, 2016) established an adaptive testing approach, which employs summary statistics from GWASs to evaluate the relationship between multiple phenotypes and a genetic variant. TATES mainly conducts *p*-values from the association between phenotypes and Single Nucleotide Polymorphisms (SNPs) and concurrently adjusting the correlations among various phenotypes (van der Sluis et al., 2013). Adaptive Fisher’s combination (AFC) (Liang et al., 2016) combines a number of optimal *p*-values from the traditional GWASs. Compared to multivariate analysis, univariate analysis is generally in a unified framework and tends to ignore the crucial information among multiple phenotypes, which may result in reducing statistical power. In recent years, a series of multivariate analysis approaches including mixed-effects models (Korte et al., 2012; Zhou and Stephens, 2014; Casale et al., 2015), Generalized Estimating Equation (GEE) (Zeger and Liang, 1986; Zhang et al., 2014), and reverse regression methods (O’Reilly et al., 2012; Yan et al., 2013; Wang et al., 2016) have been developed. Mixed-effects models comprise Linear Mixed Effects model (LME) model and Generalized Linear Mixed effects Model (GLMM), where the genetic variants are regarded as the fixed effects and the correlation among phenotypes is considered as random effects. The GEE method collapses the random effects and random residual errors in marginal regression models, which makes it different from LME. The reverse regression methods regard genotypes as the response variable and multiple phenotypes as predictors, such as the proportional odds logistic regression for joint model of multiple phenotypes (MultiPhen) (O’Reilly et al., 2012). Multivariate analysis methods are complicated, and few available software has been developed to implement these methods (Yang and Wang, 2012).

In this article, we establish a novel allele-based approach aiming at detecting association between multiple phenotypes and a genetic variant for improving the power in association studies. We first employ the Hierarchical Clustering based on Different methods for calculating Correlation coefficients (HCDC) (Fu, 2020) to cluster the enrolled phenotypes into several groups. Then, inspired by Weighted Combination of multiple Phenotypes (WCmulP) (Zhu et al., 2018), which provides optimal weights in linear combination, we perform WCmulP in each cluster to generate a novel phenotype by virtual of combining the multiple phenotypes. Subsequently, for every cluster, score test derived from the logistic regression model is constructed to test the association between the genetic variant and the novel phenotype. The permutation procedure is employed to evaluate the *p*-values of the score test statistics, and their minimum is taken as the test statistic for detecting association between the genetic variant and all phenotypes. Consequently, the Weighted Combination of multiple phenotypes following Hierarchical Clustering method (WCHC) is proposed. Using extensive simulation scenarios, we compare the performance of WCHC with that of six existing methods: O’Brien’s method (O’Brien, 1984), MultiPhen (O’Reilly et al., 2012), MANOVA (Cole et al., 1994), SHet (Zhu et al., 2015), TATES (van der Sluis et al., 2013), and WCmulP (Zhu et al., 2018). The results reveal that WCHC is either the most powerful test or comparable with the most powerful tests among the methods we compared in most of the simulation scenarios. Finally, we evaluate the performance of WCHC approach by utilizing the obesity-related phenotypes from a real dataset, Atherosclerosis Risk in Communities (ARIC) Study from dbGaP, and 11 obesity-associated SNPs are detected.

## Materials and Methods

### Proposed WCHC

Suppose a sample of *N* individuals each have *M* quantitative phenotypes *Y*_1_,*Y*_2_,…,*Y*_*M*_ and genotype *G* at a genetic variant. It is straightforward to calculate the correlation coefficient between two sets of phenotypes. Based on our previous work (Fu, 2020), the hierarchical clustering is conducted, and finally we have *K* clusters *C*_1_,*C*_2_,…,*C*_*K*_. Let *M*_*k*_ denote the number of phenotypes in the *k*th cluster *C*_*k*_, *k* = 1,2,…,*K*. We take the first cluster *C*_*1*_ as an example to show the subsequent procedure. Without loss of generality, assume *Y*_1_,*Y*_2_,…,*Y_M_1__* are the *M*_*1*_ phenotypes in the first cluster. Borrowing the allele-based regression idea (Zhu et al., 2018), we introduce *x*_*2i–1*_ = *x*_*2i*_ = 1, *x*_*2i–1*_ = *x*_*2i*_ = 0, and *x*_*2i–1*_ = 1 and *x*_*2i*_ = 0, if the genotype of the *i*th individual is *AA*, *aa*, and *Aa*, respectively, *i* = 1,2,…,*N*. By analogy, let *y*_2*i*,*j*_ = *y*_2*i*−1,*j*_ be the value of the *j*th phenotype of individual *i*, *i* = 1,2,…,*N*, *j* = 1,2,…,*M*_1_. Based on ${\left\{x_{l},y_{l,1},y_{l,2},\ldots ,y_{l,M_{1}}\right\}}_{l=1}^{2\,N}$, we establish the following model:

$$\text{logit}P\left(x_{l}=1|y_{l,1},y_{l,2},\ldots ,y_{l,M_{1}}\right)=\beta_{0}+\beta_{1}y_{l,1}+\beta_{2}y_{l,2}+\cdots +\beta_{M_{1}}\,y_{l,M_{1}},l=1,2,\ldots ,2\,N$$

to test the association between multiple phenotypes *Y*_1_,*Y*_2_,…,*Y_M_1__* and a genetic variant.

Instead of the conventional score test that is vulnerable in the case of big *M*_*1*_, we adopt the following test statistic (Zhu et al., 2018):

$$T_{1}=\sum_{l=1}^{2\,N}\left(x_{l}-\bar{x}\right)\,\left(y_{l}-\bar{y}\right),$$

where $\bar{x}=\frac{1}{2\,N}\,\sum_{l=1}^{2\,N}x_{l}$, $\bar{y}=\frac{1}{2\,N}\,\sum_{l=1}^{2\,N}y_{l}$, $y_{l}=\sum_{j=1}^{M_{1}}w_{j}\,y_{l,j}$, $w_{j}=\frac{\sum_{l=1}^{2\,N}\left(x_{l}-\bar{x}\right)\,\left(y_{l,j}-{\bar{y}}_{j}\right)}{\sum_{l=1}^{2\,N}{\left(y_{l,j}-{\bar{y}}_{j}\right)}^{2}}$, ${\bar{y}}_{j}=\frac{1}{2\,N}\,\sum_{l=1}^{2\,N}y_{l,j}$, *j* = 1,2,…,*M*_1_. Similarly, we have the corresponding test statistics *T*_2_,…,*T*_*K*_ when we study the association of the genetic variant and the multiple phenotypes in the clusters *C*_2_,…,*C*_*K*_, respectively. Further, let *p*_1_,*p*_2_,…,*p*_*K*_ be the *p*-values of *T*_1_,*T*_2_,…,*T*_*K*_, respectively, and we propose our test statistic:

$$T_{W\,C\,H\,C}=\min\left\{p_{1},p_{2},\ldots ,p_{K}\right\}.$$

As it is not easy to derive the distribution of the test statistics *T*_1_,*T*_2_,…,*T*_*K*_ under the null hypothesis of no association, the permutation procedure described below is employed to calculate the *p*-value of *T*_*WCHC*_.

(1) In each of the B permutations, we random shuffle the genotypes and then get the statistics $T_{1}^{\left(b\right)},T_{2}^{\left(b\right)}\,\ldots ,T_{K}^{\left(b\right)}$, *b* = 0,1,2,…,*B*. Note that *b* = 0 is corresponding to the original data (no permutation).

(2) Calculate $p_{k}^{\left(b\right)}$ by:

$p_{k}^{\left(b\right)}=\frac{\#\,\left\{d:T_{k}^{\left(d\right)}>T_{k}^{\left(b\right)}\,\text{for}\,d=0,1,\ldots ,B\right\}}{B}$, for *k* = 1,2,…,*K*.

and then $T_{W\,C\,H\,C}^{\left(b\right)}=\min\left\{p_{1}^{\left(b\right)},p_{2}^{\left(b\right)},\ldots ,p_{K}^{\left(b\right)}\right\}$ for *b* = 0,1,2,…,*B*;

(3) Then, the *p*-value of *T*_*WCHC*_ is given by:

$$\frac{\#\,\left\{b:\,T_{W\,C\,H\,C}^{\left(b\right)}<T_{W\,C\,H\,C}^{\left(0\right)}\,\text{for}\,b=1,2,\ldots ,B\right\}}{B}.$$

The hierarchical clustering based on our previous work (Fu, 2020) is as follows: In summary, we can find a partition ψ that partitioned *M* phenotypes into *K* disjoint clusters *C*_1_,*C*_2_,…,*C*_*K*_, where ψ = {*C*_1_,*C*_2_,…,*C*_*K*_} with ${⋃}_{k=1}^{K}C_{k}=\left\{1,2,\ldots ,M\right\}$ and *C*_*k*_⋂*C*_*l*_ = ∅(*k*≠*l*). Specifically, applying the bottom-up hierarchical clustering approach, we begin with each phenotype as a singleton cluster and then subsequently merge pairs of clusters with the largest similarity until all clusters have been merged into a single cluster that contains all phenotypes. The largest similarity in each iteration is referred as the height of the merged cluster in the dendrogram. A stopping criterion determines the number of clusters, which is similar to an established principle (Bühlmann et al., 2013). Suppose *h*_*b*_ is the largest similarity between two clusters in iteration *b* (*b* ≥ 1) or the height of iteration *b*. We define:

$$\hat{b}=\arg\,\min_{b\geq 1}\left(h_{b+1}-h_{b}\right).$$

Then, the number of clusters identified at the iteration $\hat{b}$ is chosen to determine the *K* clusters *C*_1_,*C*_2_,…,*C*_*K*_. On the calculation of correlation coefficient, the Pearson correlation coefficient, multiple correlation coefficient, and canonical correlation coefficient are respectively employed according to the number of phenotypes in the merged two clusters.

The source code for WCHC method can be found in https://github.com/YQHuFD/WCHC.

### Comparison of Methods

For convenience, let **1**_*n*_ be the all ones vector of length *n* and **0**_*n*_ be the all zeroes vector of length *n*, where *n* is a positive integer. We first list the following existing methods for power comparison with the proposed WCHC.

*OB* (O’Brien’s method) (O’Brien, 1984): Using a linear combination of univariate statistics, the OB statistic, $1_{M}^{T}\,\Sigma^{-1}\,T_{u\,n\,i}$, is developed. It is the most powerful statistic when a class of statistics is a linear combination of *T*_*uni*_, where *T*_*uni*_ is the vector of univariate statistics and **Σ** is the variance–covariance matrix of *T*_*uni*_.

*MultiPhen* (Joint model of Multiple Phenotypes) (O’Reilly et al., 2012): Modeling the genotype data as ordinal response and phenotypes as predictors, MultiPhen employs likelihood ratio test to evaluate the null hypothesis in the proportional odds logistic regression.

*MANOVA* (Multivariate ANalysis Of Variance) (Cole et al., 1994): In the standard MANOVA, there are a total of *M* phenotypes, and the *M* × *M* symmetrical background variance–covariance matrix **Σ** is unconstrained. It has ((*M* + 1) × *M*)/2 freely estimated elements in covariances and variances. Standard MANOVA tests the null hypothesis that the *M* regression coefficients are all zeroes, which is asymptotically equal to the *F*-test.

*SHet* (Test for Heterogeneous genetic effects) (Zhu et al., 2015): The test statistic of SHet, S_*Het*_, is based on S_*Hom*_, which is the most powerful statistic when the genetic effects are homogeneous. $S_{H\,o\,m}=\frac{1_{M}^{T}\,{\left(C\,o\,r\,r\,W\right)}^{-1}\,T_{u\,n\,i}\,{\left(1_{M}^{T}\,{\left(C\,o\,r\,r\,W\right)}^{-1}\,T_{u\,n\,i}\right)}^{T}}{1_{M}^{T}\,{\left(W\,C\,o\,r\,r\,W\right)}^{-1}\,1_{M}}$, where *Corr* is the correlation matrix of *T*_*uni*_, *W* is a diagonal matrix of weights for the univariate statistic. S_*Het*_ is the maximum of S_*Hom*_’s satisfying various thresholds. Specifically, only the statistics with absolute values greater than the given threshold are employed; *Corr* and *W* are partially used corresponding to the selected statistics. The *p*-value of S_*Het*_ could be estimated by simulation.

*TATES* (Trait-based Association Test that uses Extended Simes procedure) (van der Sluis et al., 2013): TATES combines the *p*-values of univariate analysis for getting a comprehensive *p*-value, while correcting the correlation between phenotypes. The TATES *p*-value is denoted as $\min\left(\frac{M_{e}\,p_{\left(j\right)}}{M_{e\,\left(j\right)}}\right)$, where *p*_(*j*)_ is the *j*^*th*^ (*j* = 1,…,*M*) sorted *p*-value in ascending order; *M*_*e*_ and *M*_*e(j)*_ denote the effective number of independent *p*-values among all *M* phenotypes and *m* specific phenotypes, respectively. The effective numbers can be obtained from the correlation matrix of *p*-values.

*WCmulP* (Weighted Combination of multiple Phenotypes) (Zhu et al., 2018): WCmulP can be taken as a component of WCHC. The original phenotypes are not used clustering and directly applied the logistic regression. Then the *T* statistic is proposed to test the association between the phenotypes and genetic variants. Lastly, the permutation procedure is used to derive the distribution of the test statistic *T*.

### Simulation Studies

Assume that the population is in Hardy–Weinberg equilibrium (HWE), and the genotypes of the genetic variants follow the binomial distribution with parameter 2 and the minor allele frequency (MAF). We set MAF = 0.3 in this simulation study for all scenarios. The multiple phenotypes are generated via the following factor model (van der Sluis et al., 2013):

$$y=\lambda \,x+c\,\gamma \,f+\sqrt{1-c^{2}}\times \varepsilon ,$$

where *y* = (*y*_1_,…,*y*_*M*_)^*T*^ is the *M* phenotypes; *x* is the genotype; λ = (λ_1_,…,λ_*M*_)^T^ is the vector of effect sizes of the variant on the *M* phenotypes; *f* is the vector of factors; *f* = (*f*_1_,…,*f*_*R*_)^*T*^∼*MVN*(0,Σ),Σ = (1−ρ)*I* + ρ*A*; *I* is the identity matrix; *A* is a matrix with elements of 1; *R* is the number of factors; and ρ is the correlation between factors; γ is an *M* × *R* matrix; *c* is a constant; ε = (ε_1_,…,ε_*M*_)^*T*^ is a vector of random errors; and ε_1_,…,ε_*M*_ are mutually independent and follow the standard normal distributions. Consider the following six models with varied numbers of factors.

Model 1: There is only one factor, and the genotype has an influence on all phenotypes with the same effect size. Namely, *R* = 1, λ=β**1**_*M*_, and γ=**1**_*M*_.

Model 2: There are two factors and a genotype has an effect on one factor with the same effect. That is, *R* = 2, $\lambda ={\left(0_{M\,/\,2}^{T},\beta \,1_{M\,/\,2}^{T}\right)}^{T}$, and γ = *bdiag*(**1**_*M*/2_,**1**_*M*/2_), which represents the block diagonal matrix of **1**_*M*/2_ and **1**_*M*/2_.

Model 3: There are two factors, and a genotype has an effect on the second factor with different sizes. That is, *R* = 2, $\lambda ={\left(0_{M\,/\,2}^{T},\frac{\beta }{M+1}{\left[1:M/2\right]}^{T}+\beta 1_{M\,/\,2}^{T}\right)}^{T}$ and γ = *bdiag*(**1**_*M*/2_,**1**_*M*/2_), where [1:*M*/2] represents the vector of components 1,2,…,*M*/2.

Model 4: There are four factors, and a genotype has an impact on the last factor with the same size. That is, *R* = 4, $\lambda ={\left(0_{3\,M\,/\,4}^{T},\beta \,1_{M\,/\,4}^{T}\right)}^{T}$, and γ = *bdiag*(**1**_*M*/4_,**1**_*M*/4_,**1**_*M*/4_,**1**_*M*/4_).

Model 5: There are four factors, and a genotype has an effect on the last factor with different sizes. Namely, *R* = 4, $\lambda ={\left(0_{3\,M\,/\,4}^{T},\frac{\beta }{M+1}{\left[1:M/4\right]}^{T}+\beta 1_{M\,/\,4}^{T}\right)}^{T}$, γ = *bdiag*(**1**_*M*/4_,**1**_*M*/4_,**1**_*M*/4_,**1**_*M*/4_).

Model 6: There are four factors, and a genotype has an impact on the last two factors with different effect directions. That is, *R* = 4, $\lambda ={\left(0_{M\,/\,2}^{T},-\frac{\beta }{M+1}{\left[1:M/4\right]}^{T}-\beta 1_{M\,/\,4}^{T},\beta 1_{M\,/\,4}^{T}\right)}^{T}$, γ = *bdiag*(**1**_*M*/4_,**1**_*M*/4_,**1**_*M*/4_,**1**_*M*/4_).

For these six models, the within-factor correlation is *c*^2^ and the between-factor correlation is ρ*c*^2^. For estimating type I error rates and powers, we fix *N* = 1,000 unrelated subjects, the number of phenotypes *M* = 16, 32. By means of setting β = 0, we generate all phenotypes that is independent of genotypes to evaluate the type I error rates of all methods, including OB, MultiPhen, MANOVA, SHet, TATES, WCmulP, and WCHC. The corresponding Q–Q plot of type I error rates is shown in Supplementary Figures 1–6. Importantly, to evaluate powers, we not only vary the values of β (while within-factor correlation *c*^2^ = 0.5 and between-factor correlation ρ*c*^2^ = 0.1) but also change the values of within-factor correlation *c*^2^ = 0.3, 0.5, 0.7, and 0.9 (while between-factor correlation ρ*c*^2^ = 0.1).

The calculation of heritability is as follows: the heritability of genotypes to the *j*-th phenotype is given by $h^{2}\,\left(y_{j}\right)=\frac{v\,a\,r\,\left(x\right)\,\lambda_{j}^{2}}{v\,a\,r\,\left(x\right)\,\lambda_{j}^{2}+1}\approx v\,a\,r\,\left(x\right)\,\lambda_{j}^{2}.$ The heritability of genotypes to the total *M* phenotypes is given by $h^{2}=\sum_{j=1}^{M}h^{2}\,\left(y_{j}\right)\approx v\,a\,r\,\left(x\right)\,\sum_{j=1}^{M}\lambda_{j}^{2}.$ Then given the parameters λ, *M*, and MAF, we can calculate *h*^2^ for the different models.

### Simulation Results

We set different nominal significance levels, various numbers of phenotypes, and distinct number of factors to estimate the type I error rates of WCHC and other six methods. For each simulation scenario, the *p*-values of WCHC, WCmulP, and SHet are evaluated by 2,000 permutations; and the *p*-values of MANOVA, MultiPhen, TATES, and OB are evaluated by their asymptotic distributions. The type I error rates of the seven methods are estimated using 2,000 replicated samples. For 2,000 replicated samples, the 95% confidence intervals (CIs) for type I error rates of nominal levels 0.01 and 0.05 are about (0.0056, 0.0144) and (0.0404, 0.0596), respectively. The evaluated type I error rates of WCHC and other six methods are presented in Table 1 (*M* = 16) and Table 2 (*M* = 32). It is observed from these two tables that most of the type I error rates of WCHC are within 95% CIs, which shows the validity of the developed WCHC. Meanwhile, the type I error rates of WCmulP, SHet, MANOVA, MultiPhen, TATES, and OB are not obviously deviated from the nominal levels. See more information in Q–Q plots (Supplementary Figures 1–6).

**TABLE 1:** Type I error rates of the seven methods in three simulation settings.

			Type I error rates			
			
	*R* = 1		*R* = 2		*R* = 4	
			
Methods	α = 0.01	α = 0.05	α = 0.01	α = 0.05	α = 0.01	α = 0.05
WCHC	0.009	0.0505	0.011	**0.065**	0.007	0.0455
WCmulP	0.0095	0.0495	0.012	0.0595	0.01	0.047
MANOVA	0.013	0.0495	0.0105	0.054	0.0075	0.0555
MultiPhen	0.014	0.0495	0.011	0.055	0.0095	0.0505
TATES	0.0105	0.0465	0.011	0.049	0.007	0.0445
SHet	0.008	0.0515	0.009	0.0535	0.0115	0.0425
OB	0.007	0.045	0.0095	0.055	0.009	0.0475

*Sample size *N* = 1,000, the number of phenotypes *M* = 16, *c*^2^ = 0.5, ρ*c*^2^ = 0.1, and minor allele frequency (MAF) = 0.3. The *p*-values of WCHC, WCmulP, and SHet are estimated by 2,000 permutations. The type I error rates of all seven method are estimated via 2,000 replicated samples at the significance of α. *R* is the number of factors. The number in bold indicates that the type I error rate is out of 95% CIs of the nominal significance level.*

**TABLE 2:** Type I error rates of the seven methods in three simulation settings.

			Type I error rates			
			
	*R* = 1		*R* = 2		*R* = 4	
			
Methods	α = 0.01	α = 0.05	α = 0.01	α = 0.05	α = 0.01	α = 0.05
WCHC	0.0095	0.0525	0.0105	0.0525	0.0105	0.0565
WCmulP	0.013	0.0545	0.0135	0.051	0.01	0.05
MANOVA	**0.0175**	**0.072**	0.0115	0.059	0.009	0.0535
MultiPhen	**0.0155**	**0.072**	0.0115	0.054	0.01	0.055
TATES	0.0105	0.048	0.0115	0.0475	0.011	0.049
SHet	0.0125	**0.061**	0.0095	0.0475	**0.005**	0.0415
OB	0.011	0.0555	0.0135	0.0555	0.01	0.044

*Sample size *N* = 1,000, the number of phenotypes *M* = 32, *c*^2^ = 0.5, ρ*c*^2^ = 0.1, and minor allele frequency (MAF) = 0.3. The *p*-values of WCHC, WCmulP, and SHet are estimated by 2,000 permutations. The type I error rates of all seven method are estimated via 2,000 replicated samples at the significance of α. *R* is the number of factors. The number in bold indicates that the type I error rate is out of 95% CIs of the nominal significance level.*

**FIGURE 1:**
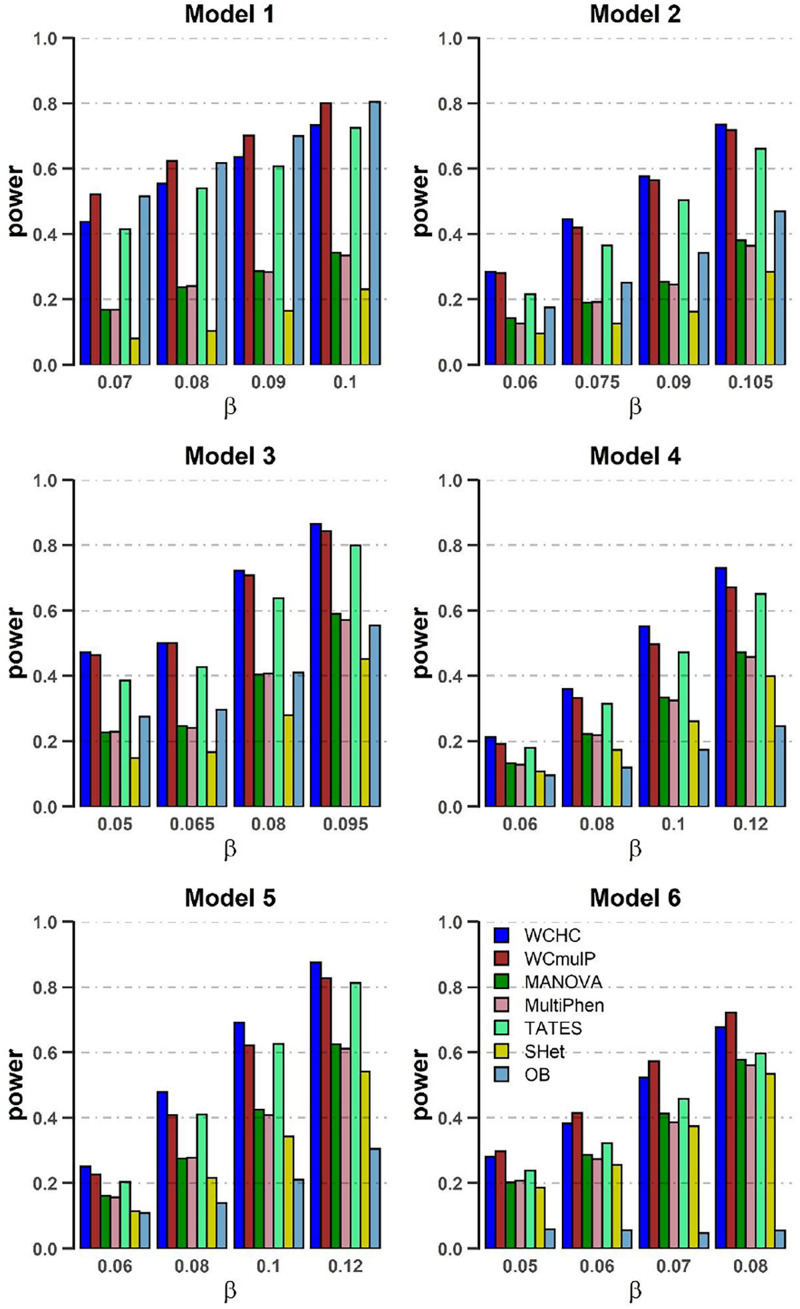
Power comparisons of the seven methods as a function of β in the six models. Sample size is *N* = 1,000, the number of phenotypes is *M* = 16, *c*^2^ = 0.5, ρ*c*^2^ = 0.1, and MAF = 0.3. The power of all the seven methods is estimated by 1000 replicated samples at a significance level of 0.05.

**FIGURE 2:**
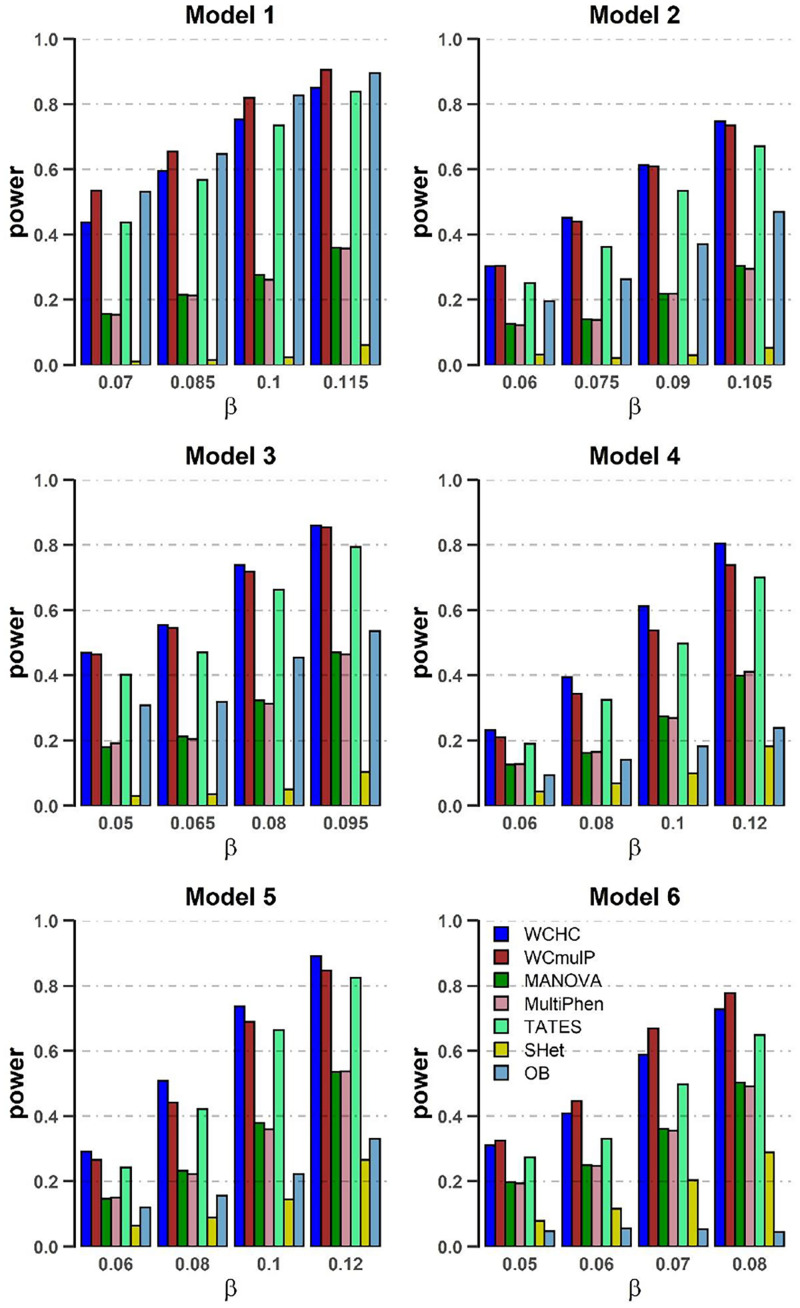
Power comparisons of the seven methods as a function of β in the six models. Sample size is *N* = 1,000, the number of phenotypes is *M* = 32, *c*^2^ = 0.5, ρc^2^ = 0.1, and MAF = 0.3. The power of all the seven methods is estimated by 1000 replicated samples at a significance level of 0.05.

**FIGURE 3:**
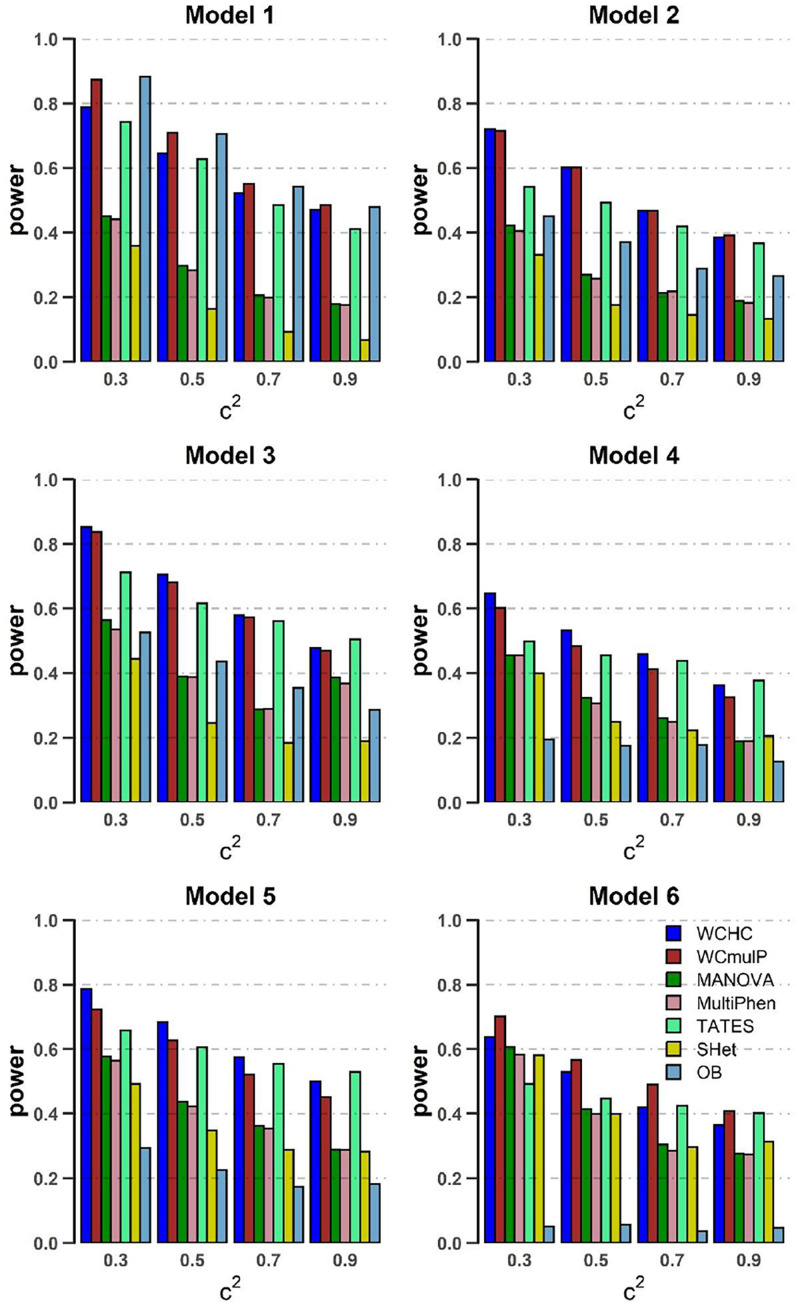
Power comparisons of the seven methods as a function of *c*^2^ in the six models. Sample size is *N* = 1,000, the number of phenotypes is *M* = 16, ρc^2^ = 0.1 and MAF = 0.3. β = 0.09 for model 1 and 2; β = 0.08 for model 3; β = 0.1 for model 4 and 5; β = 0.07 for model 6. The power of all the seven methods is estimated by 1000 replicated samples at a significance level of 0.05.

**FIGURE 4:**
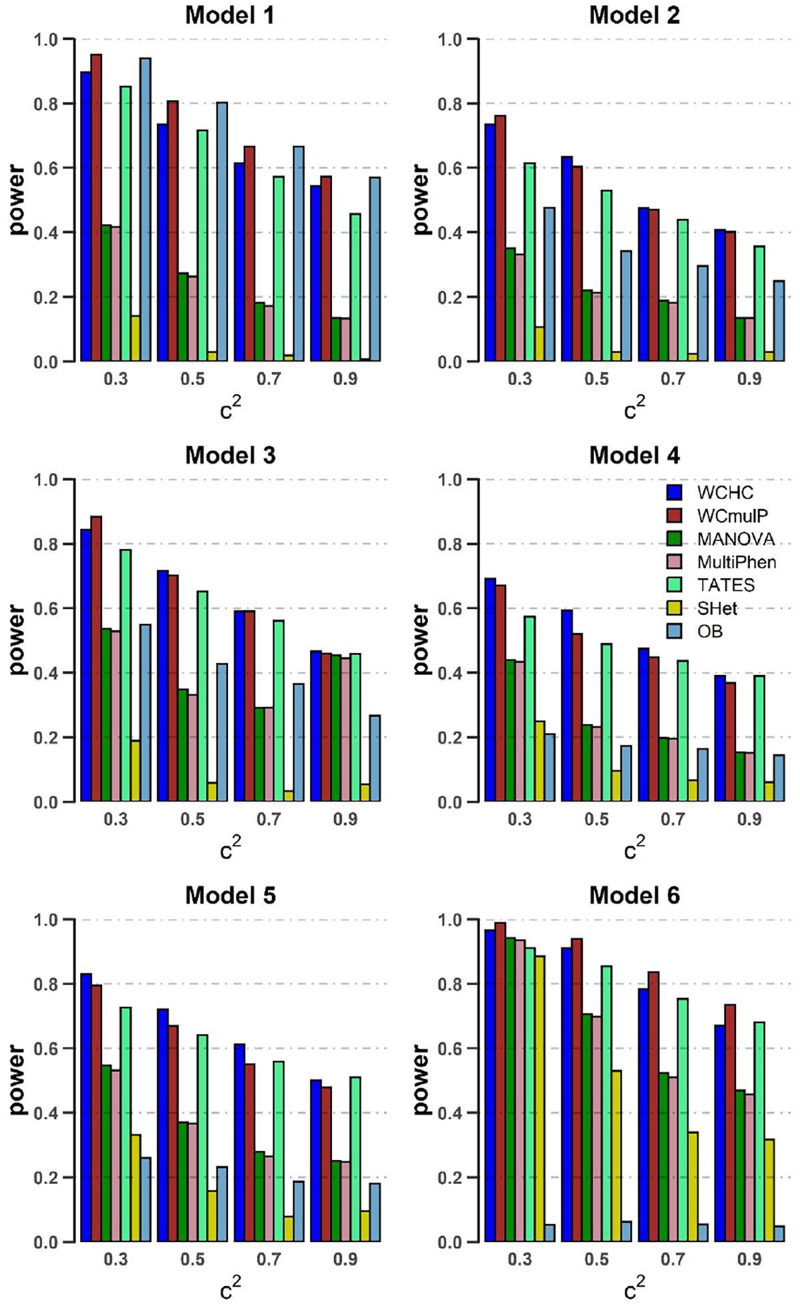
Power comparisons of the seven methods as a function of *c*^2^ in the six models. Sample size is *N* = 1,000, the number of phenotypes is *M* = 32, ρ*c^2^* = 0.1, and MAF = 0.3. β = 0.1 for model 1 and 4–6; β = 0.09 for model 2; β = 0.08 for model 3. The power of all the seven methods is estimated by 1000 replicated samples at a significance level of 0.05.

In order to compare powers of these seven methods, we plot power against the genetic effect β (in Figures 1, 2) and the within-factor correlation *c*^2^ (in Figures 3, 4). Note in the calculation of power, the *p*-values of WCHC, WCmulP, and SHet are evaluated by 1,000 permutations; the powers of the seven methods are estimated based on 1,000 replicated samples at a significance level of 0.05. The following observations can be drawn from the simulation.

(1) As expected, in each model, the powers of all seven methods increase as the genetic effect β increases (see Figures 1, 2). (2) Except in models 1 and 6, WCHC is the most powerful test in all the methods under most of the simulation scenarios (see Figures 1–4). (3) As the number of phenotypes increases from *M* = 16 to *M* = 32, WCHC exhibits more obvious advantages over other methods except in Model 1 and 6 (see Figures 1, 2). (4) No matter changes of genetic effects β or variations of correlation coefficients between different phenotypes, MANOVA and MultiPhen have the similar performance in all six models. (5) Generally, in each model, the power of all methods decreases with the increase of correlation coefficients of within factors between phenotypes. (6) OB is the most powerful test when the genetic effects are homogeneous (Model 1). However, OB’s power decreases when there exist opposite directions (Model 6) or when the genetic variant has an influence on a small proportion of phenotypes (Model 5). (7). In general, WCHC, WCmulP, and TATES are more powerful than SHet, OB, MANOVA, and MultiPhen when the genetic variant affects a portion of phenotypes (Models 2–6). (8). WCHC shows obvious advantages over other methods when the genetic variant only affects part of the phenotypes with the same direction. One possible reason is that in the models of generating data, the genetic variant has effects of the same directions on some phenotypes and has no effect on the remaining ones. The hierarchical clustering is capable of grouping similar phenotypes together, so as to reduce the dimensions of association test for improving the power to detect the associated phenotypes.

Overall, from all the power simulation results, we could draw that our proposed WCHC has advantages over other methods in most scenarios, and especially in some scenes, the ascendancy is obvious. In other scenarios, WCHC is comparable with the most powerful test.

## Real Data Analysis

We applied our proposed method WCHC to the real data analysis from ARIC study (see more details in The ARIC Investigators, 1989). In brief, sponsored by the National Heart, Lung, and Blood Institute (NHLBI), ARIC is a prospective cohort study of atherosclerosis risk in community. It records the changes of the incidence of atherosclerosis-related diseases and cardiovascular risk factors in distinct races, regions, genders, and time, aiming at investigating the etiology and natural process of atherosclerosis (Morrison et al., 2013). We obtain the genotyped and clinical phenotypic data in ARIC from dbGaP server of the United States National Center for Biotechnology Information (accession number: phs000090.v4.p1).

To evaluate the performance of WCHC in real data, we use the seven methods to analyze obesity-related phenotypes in ARIC. We selected nine continuous traits with regard to obesity including weight, body mass index (BMI), average skinfold thickness of the triceps brachii, mean subscapular skinfold thickness, waist, hip girth, waist-to-hip ratio, calf girth, and wrist breadth and three covariates including age, gender, and race. The specific description of these variables is listed in Table 3, and the correlation structure of obesity-related phenotypes is given in Supplementary Figure 7. A set of 12,701 subjects across 272,027 SNPs were left for subsequent analysis after excluding subjects with missing data in any of the 12 variables as well as the genetic variants with missing rate greater than 0.2 or HWE < 10^–4^. Every phenotype is adjusted for those three covariates using linear regression model.

**TABLE 3:** Summary statistics of obesity-related indexes and covariates in ARIC.

Index	All	Gender			Race		
			
		Male	Female	*p*-value	White	Black	*p*-value
*N*	12,771	5,704	7,067	–	9,633	3,138	–
Male, %	44.66	–	–	–	47.02	37.44	**9.11 × 10^–21^**
Age, years	54.09 ± 5.73	54.450 ± 5.75	53.76 ± 5.69	**6.76 × 10**^–^**^13^**	54.34 ± 5.68	53.34 ± 5.80	**5.51 × 10**^–^**^17^**
Weight, lb	173.13 ± 36.85	188.27 ± 31.46	160.92 ± 36.36	**<2.2 × 10**^–^**^16^**	169.61 ± 35.69	183.99 ± 38.25	**1.90 × 10**^–^**^74^**
Weight missing, %	0.149	0.158	0.142	0.995	0.083	0.351	**0.002**
BMI, kg/m^2^	27.66 ± 5.30	27.54 ± 4.18	27.75 ± 6.05	**0.020**	27.01 ± 4.86	29.65 ± 6.05	**9.98 × 10**^–^**^104^**
BMI missing, %	0.149	0.158	0.142	0.995	0.083	0.351	**0.002**
Triceps, mm	25.26 ± 10.02	19.34 ± 7.87	30.04 ± 8.97	**<2.2 × 10**^–^**^16^**	24.54 ± 9.08	27.48 ± 12.23	**1.73 × 10**^–^**^34^**
Triceps missing, %	0.157	0.175	0.142	0.798	0.093	0.351	**0.004**
Scapular, mm	24.48 ± 11.59	22.22 ± 9.19	26.31 ± 12.92	**1.13 × 10**^–^**^94^**	21.85 ± 9.33	32.59 ± 13.89	**1.60 × 10**^–^**^299^**
Scapular missing, %	0.446	0.561	0.354	0.107	0.353	0.733	**0.009**
WC, cm	96.94 ± 13.83	99.23 ± 10.93	95.09 ± 15.54	**1.25 × 10**^–^**^68^**	96.19 ± 13.33	99.25 ± 15.02	**5.34 × 10**^–^**^24^**
WC missing, %	0.141	0.123	0.156	0.798	0.104	0.255	0.092
HC, cm	104.55 ± 10.31	102.85 ± 8.09	105.93 ± 11.63	**2.81 × 10**^–^**^68^**	103.50 ± 9.478	107.79 ± 11.98	**7.52 × 10**^–^**^72^**
HC missing, %	0.141	0.140	0.142	0.999	0.104	0.255	0.092
WHtR	0.926 ± 0.078	0.963 ± 0.054	0.895 ± 0.081	**<2.2 × 10**^–^**^16^**	0.928 ± 0.079	0.920 ± 0.076	**4.66 × 10**^–^**^8^**
WHtR missing, %	0.149	0.140	0.156	0.999	0.114	0.255	0.131
Calf, cm	37.44 ± 3.67	38.06 ± 3.17	36.95 ± 3.95	**1.48 × 10**^–^**^68^**	37.39 ± 3.58	37.60 ± 3.93	**0.006**
Calf missing, %	0.157	0.210	0.113	0.248	0.114	0.287	0.062
Wrist, mm	53.62 ± 5.18	57.78 ± 3.66	50.27 ± 3.53	**<2.2 × 10**^–^**^16^**	53.59 ± 5.26	53.74 ± 4.91	0.137
Wrist missing, %	0.117	0.123	0.113	0.999	0.073	0.255	**0.022**

**N* is the number of subjects; BMI is body mass index; Triceps is average skinfold thickness of the triceps brachii; Scapular is mean subscapular skinfold thickness; WC is waist; HC is hip girth; WHtR is waist-to-hip ratio; Calf is calf girth; and Wrist is wrist breadth. The distributions of normal index is described by mean ± standard deviation; the distributions of non-normal indicators are described by means (25% quantile, 75% quantile). For normal distribution indicators, the differences between groups are evaluated using the *t*-test (the variances of two groups are homogeneous) or the approximate *t*-test (the variances of two groups are heterogeneous). For non-normally indicators, Wilcoxon signed rank test is used to test the differences between indicators to get the *p*-values of differences. For discrete indicators, the chi-square test is used for hypothesis testing and then deriving *p*-values. Bold number indicates *p* < 0.05. ARIC, Atherosclerosis Risk in Communities.*

Based on these adjusted phenotypes related to obesity, we employ WCHC and other six methods to detect associated SNPs. Two groups are obtained after clustering the nine phenotypes in the real data analyses by the hierarchical clustering in WCHC. One of the clusters only includes wrist breadth, while the other encompasses the remaining phenotypes. Because of multiple testing correction, we adopt the significance threshold of 1 × 10^–7^, not the traditional genome-wide significance threshold of 5 × 10^–8^. There are totally 11 SNPs that are significant for at least one method (Table 4). Previous studies (Frayling et al., 2007; Heard-Costa et al., 2009; Lindgren et al., 2009; Meyre et al., 2009; Thorleifsson et al., 2009; Willer et al., 2009; Heid et al., 2010; Speliotes et al., 2010; Bradfield et al., 2012; Wen et al., 2012; Berndt et al., 2013; Monda et al., 2013; Locke et al., 2015; Shungin et al., 2015) have reported that *FTO* leads to obesity through population studies and experimental researches elaborating relevant mechanisms. Among the 11 identified SNPs, rs9939609 and rs8050136 are involved in *FTO*. Additionally, rs7968682 is reported to be associated with height (Yang T. L. et al., 2010; Takeshita et al., 2011). Few other SNPs have been assessed to explore the association with obesity or obesity-related phenotypes. From Table 4, we can see that both WCHC and MANOVA identified six SNPs; TATES identified five SNPs; both WCmulP and SHet identified four SNPs; MultiPhen identified three SNPs; and OB only identified one SNP, which may be due to that the true genetic effects of most of SNPs are heterogeneous for all phenotypes. In summary, the number of SNPs identified by WCHC is comparable with the largest number of SNPs identified by other tests. These real data analysis results are consistent with our simulation results.

**TABLE 4:** Significant SNPs and the corresponding *p*-values in the analysis of ARIC.

Chr	SNP	OB	MultiPhen	MANOVA	SHet	TATES	WCmulP	WCHC
3	rs17017947	**1.57 × 10**^–^**^12^**	NA	**1.02 × 10**^–^**^11^**	**0**	0.314	0.513	0.672
10	rs41470552	0.062	NA	**6.25 × 10**^–^**^9^**	1.15 **×** 10^–4^	0.035	0.078	0.141
11	rs7927943	0.099	3.33 × 10^–6^	5.57 × 10^–6^	8.00 × 10^–7^	**1.16 × 10**^–^**^8^**	**1 × 10**^–^**^7^**	**1.00 × 10**^–^**^7^**
11	rs1945647	0.038	6.27 × 10^–6^	1.2 × 10^–5^	7.00 × 10^–7^	**1.77 × 10**^–^**^8^**	**0**	**1.00 × 10**^–^**^7^**
11	rs7114045	3.73 × 10^–5^	5.47 × 10^–6^	**5.66 × 10**^–^**^8^**	0.003	0.018	0.051	0.108
12	rs7968682	0.414	**5.36 × 10**^–^**^8^**	**8.34 × 10**^–^**^8^**	**0**	0.018	0.079	0.006
16	rs9939609	0.082	**3.39 × 10**^–^**^8^**	**1.85 × 10**^–^**^8^**	**0**	**2.97 × 10**^–^**^10^**	**0**	**1.00 × 10**^–^**^7^**
16	rs8050136	0.186	**8.66 × 10**^–^**^8^**	**4.29 × 10**^–^**^8^**	**0**	**2.86 × 10**^–^**^9^**	**0**	**1.00 × 10**^–^**^7^**
20	rs201561	0.138	2.91 × 10^–6^	2.48 × 10^–6^	6.30 × 10^–6^	7.99 × 10^–7^	0.035	**1.00 × 10**^–^**^7^**
20	rs1570004	0.184	7.77 × 10^–5^	5.28 × 10^–5^	1.90 × 10^–6^	**6.12 × 10**^–^**^8^**	0.001	**1.00 × 10**^–^**^7^**
20	rs1014883	0.457	3.00 × 10^–5^	1.83 × 10^–5^	1.60 × 10^–6^	3.19 **×** 10^–4^	0.011	1.07 **×** 10^–4^

*The *p*-values of WCHC, SHet, and WCmulP are estimated using 10^7^ permutations; the *p*-values of OB, MultiPhen, MANOVA, and TATES are estimated using asymptotic distributions; and the bold number indicates *p*-value ≤ 1 × 10^–7^. HWE ≥ 10^–4^ for the identified SNPs; “NA” indicates MultiPhen is not available, as the genotype at the specified SNP does not take all three values of 0, 1, and 2 in these data. **0** indicates *p*-value < 10^–7^. SNP, single-nucleotide polymorphism; ARIC, Atherosclerosis Risk in Communities; HWE, Hardy–Weinberg equilibrium.*

## Characteristics of the Significant Variants

Table 5 shows the annotations of the identified SNPs based on the Ensemble website^1^ and SCAN website^2^. From Table 5, we can see that the significant SNPs are located in intergenic or intron region, and most of them have been reported to be associated with BMI, height, or T2D. Generally, they have been reported in GWAS. We could also explore the expression of genes associated with the significant SNPs, although they are located in intergenic or intron region. Therefore, we make full use of Qtlizer^3^, eQTLGen^4^, and PsychENCODE^5^, which are the largest integrating various tissues, blood, and brain expression Quantitative Trait Locus (eQTL) samples, respectively. Instead of restricting analysis to the SNPs in Table 5, we considered using a larger list of SNPs with proxy variants, which are in Linkage Disequilibrium (LD) with the SNPs in Table 5 (*r*^2^ ≥ 0.8) via Qtlizer website. We restricted the eQTL association criteria with False Discovery rate (FDR) < 0.05. The results of eQTLs in Qtlizer, eQTLGen, and PsychENCODE are displayed in Supplementary Data Sheets.

**TABLE 5:** Characteristics of the significant SNPs.

SNPs	Chr.	Position (GRCh38)	Alleles (Alt/Ref)	Gene (nearest)	Feature	Expression genes	Reported (yes/no)	Reported phenotypes	GWAS references
rs17017947	3	276,171	A/C	*CHL1*	Intron	–	No	–	–
rs41470552	10	102,222,133	T/G	*PITX3*	Intergenic	–	No	–	–
rs7114045	11	101,479,689	C/T	*TRPC6*	Intron	–	No	–	–
rs1945647	11	81,602,715	C/T	*MTND6P25*	Intergenic	*GNAI2*, *STK40*, *LIMK1*, *LIG4*, *HLTF*, *ZNF511*, *CBLL1*, *NUDT17*, *POLR3C*, *DAGLB*, *KDELR2*, *NUP93*, *PRCC*, *C16orf80*, *RAB33B*, *LRP8*	No	–	–
rs7927943	11	81,637,194	C/T	*MTND6P25*	Intergenic	*WSCD2*, *GNAI2*, *ZFHX3*, *NUP93*, *FAM60A*, *LIMK1*, *MAP4*, *FLJ31958*, *LIG4*, *HLTF*	No	–	–
rs7968682	12	65,978,100	G/T	*HMGA2*	Intergenic	–	Yes	Height, birth weight	
rs8050136	16	53,782,363	C/A	*FTO*	Intron	*HES7*, *LATS2*	Yes	BMI, T2D, adiposity	
rs9939609	16	53,786,615	T/A	*FTO*	Intron	*CR1*, *CR1L*, *ZNRF1*, *ANKRD50*, *LATS2*, *TSPYL4*, *HES7*	Yes	BMI, T2D	
rs1014883	20	21,863,992	A/G	*RPL41P1*	Intergenic	*ANTXR2*	No	–	–
rs1570004	20	35,370,450	A/T	*UQCC*	Intron	–	Yes	Height	–
rs201561	20	22,018,575	G/C	*RPL41P1*	Intergenic	*P2RX3*, *EHD4*	Yes	Balding type 1	

*Annotations are from Ensemble website (https://asia.ensembl.org) and SCAN website (http://scandb.org); intron denotes the SNP is located between exons; intergenic denotes the SNP is located between genes. Expression genes denotes annotations added after analysis of transcriptional levels of eQTL in cell lines from HapMap CEU and YRI samples using Affymetrix human exon 1.0 ST array. GWAS references refer to the identifications of PubMed. SNP, single-nucleotide polymorphism; GWAS, genome-wide association study; eQTL, expression quantitative trait locus.*

**FIGURE 5:**
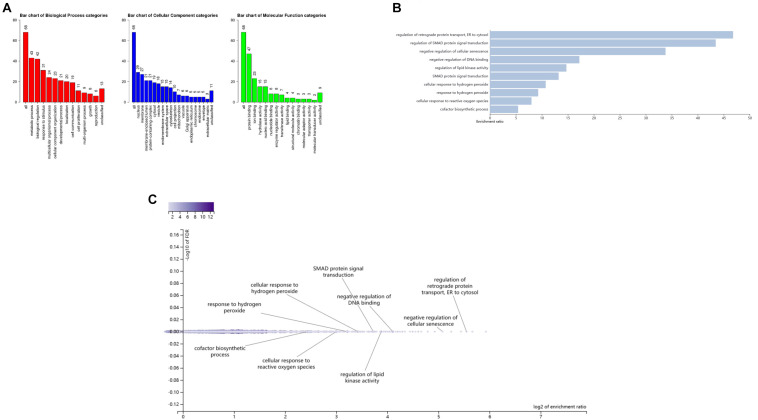
GO enrichment analysis of significant SNPs probability regulating associated genes expression. **(A)** Red, blue, and green bars indicate biology progress, cellular components, and molecular function categories, respectively. The numbers above the bar charts indicate the number of genes in each of the biological categories; **(B)** Bar charts of GO enrichment analysis; **(C)** Volcano plot of GO enrichment analysis. For more knowledge about GO enrichment, please check the website http://geneontology.org/docs/go-enrichment-analysis/.

**FIGURE 6:**
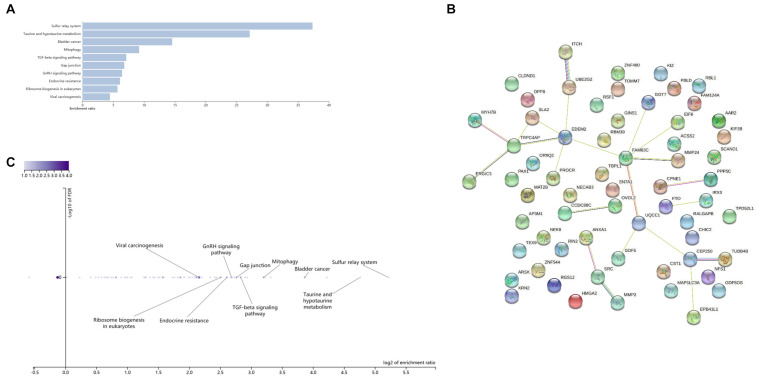
KEGG enrichment analysis and PPI network diagram of significant SNPs probability regulating associated genes expression. **(A)** Bar chats of KEGG enrichment analysis; **(B)** Volcano plot of KEGG enrichment analysis; **(C)** PPI interaction network diagram, data are from https://www.string-db.org/.

In order to further study the biology function of the genetic variants, we performed enrichment analysis on genes associated with these 11 SNPs in Table 5 and the proxy variants (see qtlizer.results in Supplementary Data Sheet) in the three websites/consortiums (Qtlizer, eQTLGen, and PsychENCODE). After summarizing all genes in the three tables (see qtlizer.results, eQTLGen.results, and PsychENCODE.results in Supplementary Data Sheet), we got all the genes associated with the eQTLs (see Gene sheet in Supplementary Data Sheet). A total of 76 genes were obtained to do the gene set analysis by virtue of different biological databases for investigating biological processes, cell components, molecular functions, metabolic pathways, phenotypes with relevant diseases, and protein interactions. The results of enrichment analysis and protein–protein interaction (PPI) are given in Figures 5, 6. According to the Gene Ontology (GO) enrichment analysis chart in Figure 5, GO items mainly focus on the cellular response to hydrogen and regulation of lipid kinase activity, which may be parts of the metabolic process. Moreover, the Kyoto Encyclopedia of Genes and Genomes (KEGG) metabolic pathway in Figure 6 presents that the enriched genes possess taurine and hypotaurine metabolism and endocrine resistance, which indicates that the obesity-related variants detected by WCHC and other methods in ARIC might be involved in the metabolic pathways, which releases the signal that our results are in a certain degree of credibility. Subsequently, we draw a PPI network diagram through the STRING^6^ to reveal that most of the proteins (nodes) encoded by the genes have certain interactions (edges), which suggests the proteins related the expression of genes might interact with each other for controlling a variety of biological phenomena including endocrine development, cellular response to hydrogen, and metabolic processes.

Overall, our results showed that WCHC and other six methods could identify significant genetic variants for obesity phenotypes in real data analysis from ARIC. More importantly, functional annotations of genetic variants and enrichment analysis support that the variants are closely related to biological functions and metabolic pathways of obesity.

## Discussion

In this article, we proposed WCHC to perform multivariate analysis of multiple phenotypes in association studies due to the following reasons. (1) Multiple correlated phenotypes are usually measured in complex disease for genetic association studies. Compared to univariate analysis, multivariate analysis considers multidimensional structure information. It indicates certain variance–covariance is included in multiple phenotypes. (2) Association analysis of multiple phenotypes separately cannot present genetic interactions between phenotypes. More and more evidence reveals that joint analysis of multiple related phenotypes, which considers the interactions between phenotypes comprehensively, can boost the power of detecting genetic variants associated with complex diseases. No matter whether the effects of genetic variants on phenotypes are consistent or not, WCHC provides a relatively simple way to incorporate the correlations between phenotypes into analysis. (3) Actually, we are not sure which phenotype or linear combination of phenotypes is more likely to elucidate the genetic structure of complex diseases. WCHC adopts clustering approach and linear combination of multiple phenotypes to account for the complex genetic information, which not only takes the similarity between phenotypes into consideration but also considers the heterogeneity, so it is helpful to explore the genetic mechanism of diseases.

Our results manifested that WCHC has correct type I error rates and is either the most powerful test or comparable with the most powerful tests among the seven methods we adopted. None of the other methods observes consistently good performances under the simulation scenarios. OB is the most powerful test when the genetic effects are homogeneous, while it loses power dramatically when genetic effects are heterogeneous, especially if there exists opposite directions of genetic effects. In most simulation scenarios, SHet, MANOVA, MultiPhen, and TATES have similar powers, and they are less powerful than WCHC, and WCHC is more powerful when the genetic variant influences a part of phenotypes. However, WCmulP is less powerful in this scenario. Furthermore, in real data analysis, WCHC and MANOVA identified the largest number of significant SNPs (six SNPs). Therefore, the real data analysis results demonstrate that WCHC has excellent performance in detecting SNPs associated with complex disease with multiple related phenotypes such as obesity. As for the methods giving such different results when applied to the real ARIC data, we think that the parametric information of real data is unknown for us. Therefore, we may try various methods to analyze the real data for getting reliable results as much as possible. In our opinion, no method can guarantee 100% accuracy. We can only be cautious to say that the significant loci are more likely to be true signals, but further verification is still needed.

In the context of association studies, population stratification (PS) refers to allele frequency difference between populations uncorrelated to the outcome of interest, but due to systematic ancestry differences. PS may cause confounding effects seriously if not adjusted properly (Knowler et al., 1988; Lander and Schork, 1994). Methods such as principal component analysis (PCA) (Zhu et al., 2002; Chen et al., 2003; Zhang et al., 2003; Price et al., 2006; Bauchet et al., 2007), linear mixed model (LMM) (Kang et al., 2010; Zhang et al., 2010; Hoffman, 2013), multidimensional scaling (MDS) (Li and Yu, 2008), robust PCA based on resampling by half means (RPCA-RHM) (Liu et al., 2013), and robust PCA based on the projection pursuit (RPCA-PP) (Liu et al., 2013) can be used to adjust for PS. We propose to apply PCA to control for PS when samples from different populations are involved.

In real data analysis, as the number of phenotypes elevates, the chance of missing at least one subject increases exponentially, especially in epidemiological and clinical research (Ali et al., 2011; Dahl et al., 2016). We removed 412 subjects with missing either phenotypes or covariates from 13,113 observations. It is worth noting that the sample mean substitution (Ali et al., 2011; van der Sluis et al., 2013) is a simple, unconditional method that does not depend on other variables, which is a common strategy replacing the missing values with plausible values for the variable with missing values. However, it may contribute to biased estimates where data are not missing completely at random (Ali et al., 2011). Additionally, imputation is a more complicated approach that fills in missing values with estimated values via model-based methods or conditional imputation, comprising multiple imputation (MI), multivariate normal imputation (MVNI), and fully conditional specification (FCS) (Raghunathan et al., 2001; Buuren et al., 2006; De Silva et al., 2017).

One weakness of WCHC is that the test statistic does not have an asymptotic distribution and its *p*-value needs to be calculated by permutation procedure, which is time-consuming as compared with approaches whose test statistics have asymptotic distributions. To conduct GWAS, a small number of permutations (e.g., 1,000) can be used to select genetic variants that reveal evidence of association, and then a large number of permutations are employed to estimate the selected significant genetic variants. We adopted this strategy to analyze the real dataset. Consequently, it seems to be efficient, and the bioinformatics analysis of significant variants supports our results. In conclusion, in the field of genotype–phenotype association studies, WCHC is an effective method for association analysis of multiple phenotypes, which considers both the correlations and differences among the multiple phenotypes. WCHC provides a convenient approach of association analysis for researchers to discover potential genes causing complex diseases, which does not need to assume the genetic model, and there is no limit to the number of phenotypes. Because the genetic structure of phenotypes is usually unknown, WCHC provides a convenient statistical method for the application of massive multi-phenotypic data in the future.

## Data Availability Statement

Publicly available datasets were analyzed in this study. This data can be found here: phs000090.v4.p1. The source code for WCHC method can be found in https://github.com/YQHuFD/WCHC.

## Ethics Statement

The studies involving human participants were reviewed and approved by the ARIC. The patients/participants provided their written informed consent to participate in this study.

## Author Contributions

LF and Y-QH: study concept and design and drafting of the manuscript. LF, Y-QH, and YW: acquisition of data. LF: methodology and interpretation of data. LF, YW, TL, and Y-QH: critical revision of the manuscript for important intellectual content. All authors have read and approved the final version of manuscript. We thank all reviewers and editors for their valuable suggestions on revision.

## Conflict of Interest

The authors declare that the research was conducted in the absence of any commercial or financial relationships that could be construed as a potential conflict of interest.

## Footnotes

1 https://asia.ensembl.org

2 http://scandb.org

3 http://genehopper.de/qtlizer

4 https://www.eqtlgen.org/

5 http://resource.psychencode.org/

6 https://www.string-db.org/
