# Supplementary material for: A Novel Approach Integrating Hierarchical Clustering and Weighted Combination for Association Study of Multiple Phenotypes and a Genetic Variant
Source: Front Genet. 2021 Jun 17;12:654804. doi: 10.3389/fgene.2021.654804 (PMC8249926; doi:10.3389/fgene.2021.654804)
Supplement: Supplementary file 2 [file Data_Sheet_2.pdf]

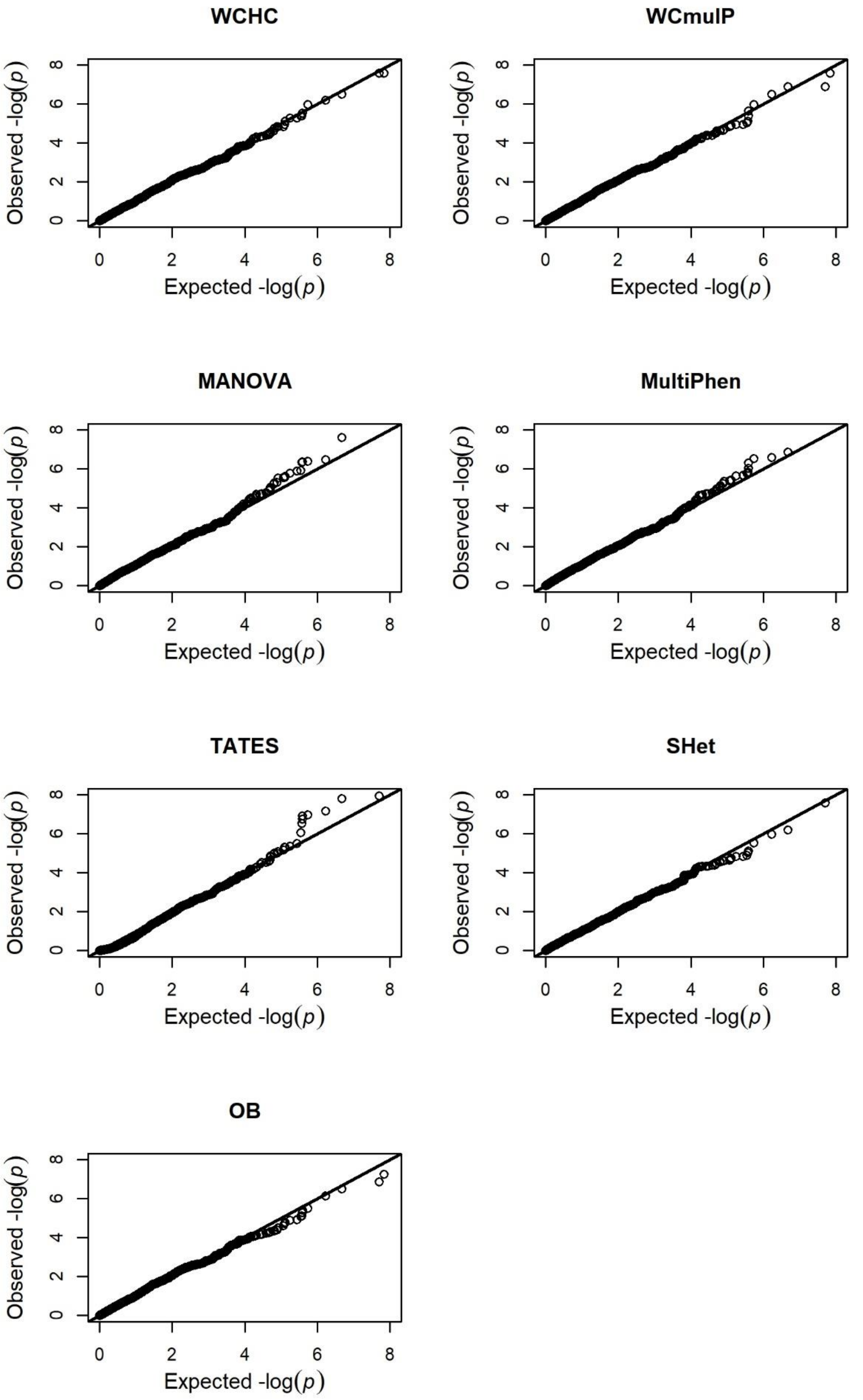

Supplementary Figure 1. Type I error rate comparisons of the seven methods in Q-Q plot. Sample size is $N = 1000$, the number of phenotypes is $M = 16$, the number of factors is $R = 1$, $c^2 = 0.5$, $\rho c^2 = 0.1$, and MAF = 0.3. The type I error rate of all the seven methods is estimated using 2000 replicated samples.

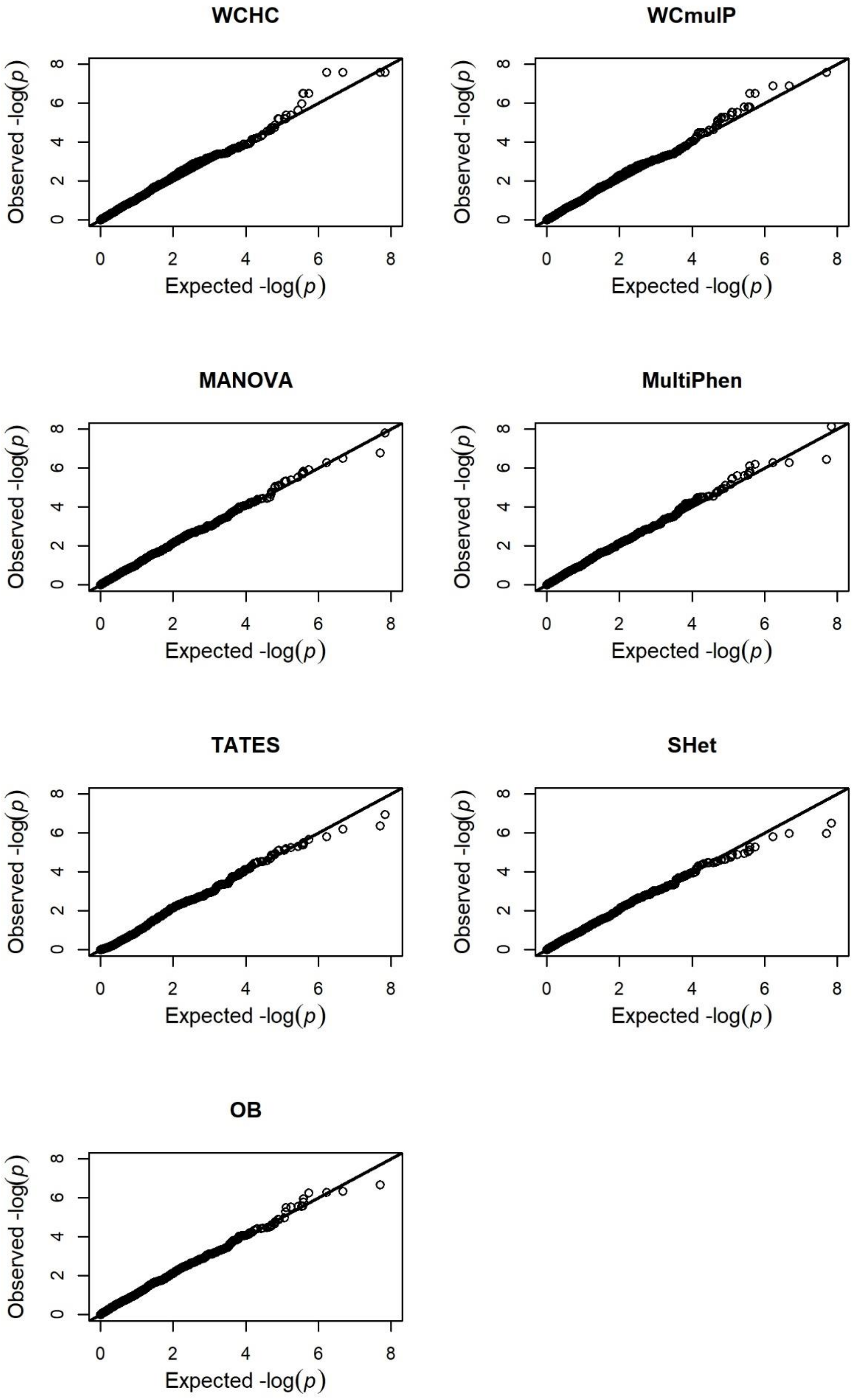

Supplementary Figure 2. Type I error rate comparisons of the seven methods in Q-Q plot. Sample size is $N = 1000$, the number of phenotypes is $M = 16$, the number of factors is $R = 2$, $c^2 = 0.5$, $\rho c^2 = 0.1$, and MAF = 0.3. The type I error rate of all the seven methods is estimated using 2000 replicated samples.

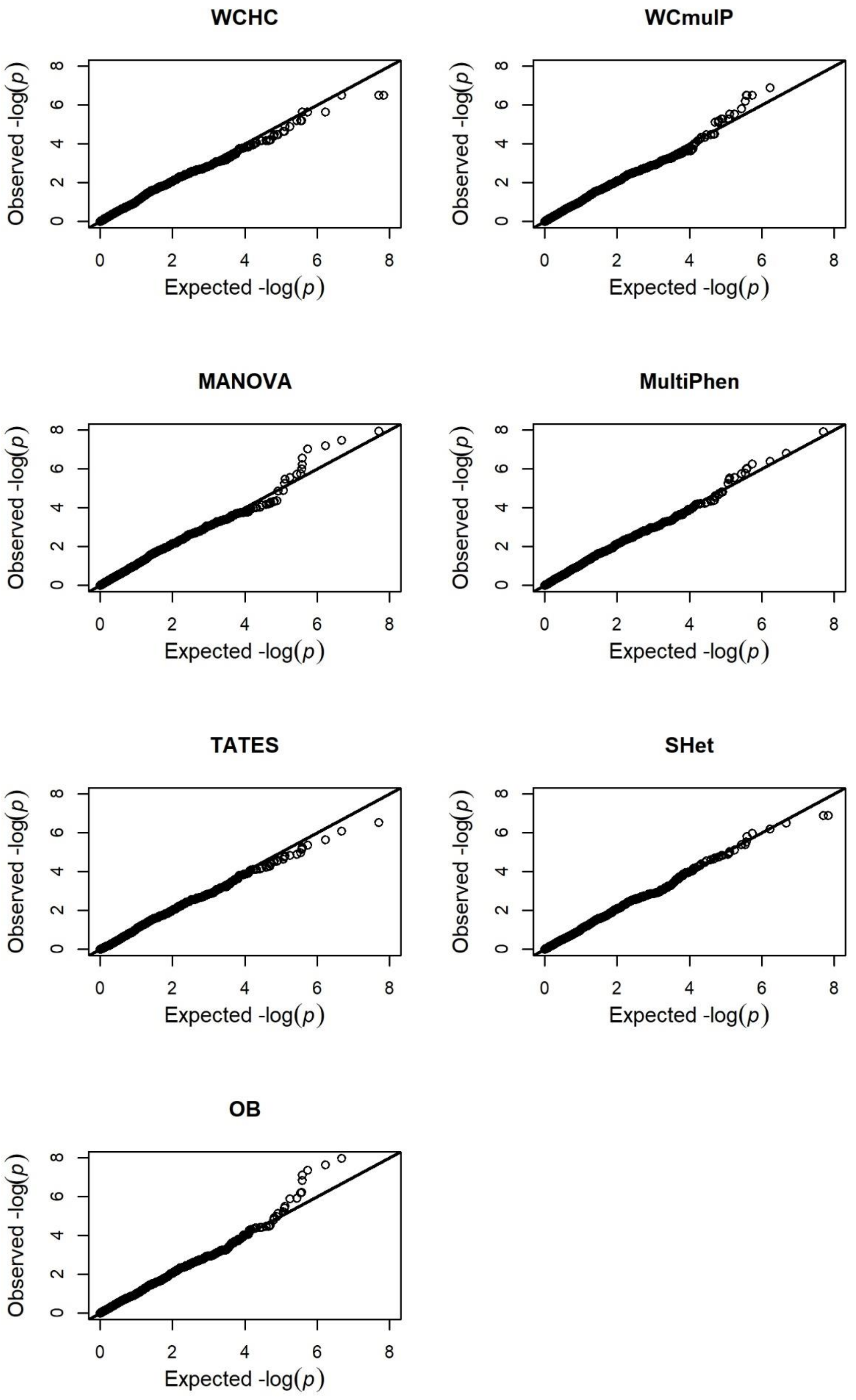

Supplementary Figure 3. Type I error rate comparisons of the seven methods in Q-Q plot. Sample size is $N = 1000$, the number of phenotypes is $M = 16$, the number of factors is $R = 4$, $c^2 = 0.5$, $\rho c^2 = 0.1$, and MAF = 0.3. The type I error rate of all the seven methods is estimated using 2000 replicated samples.

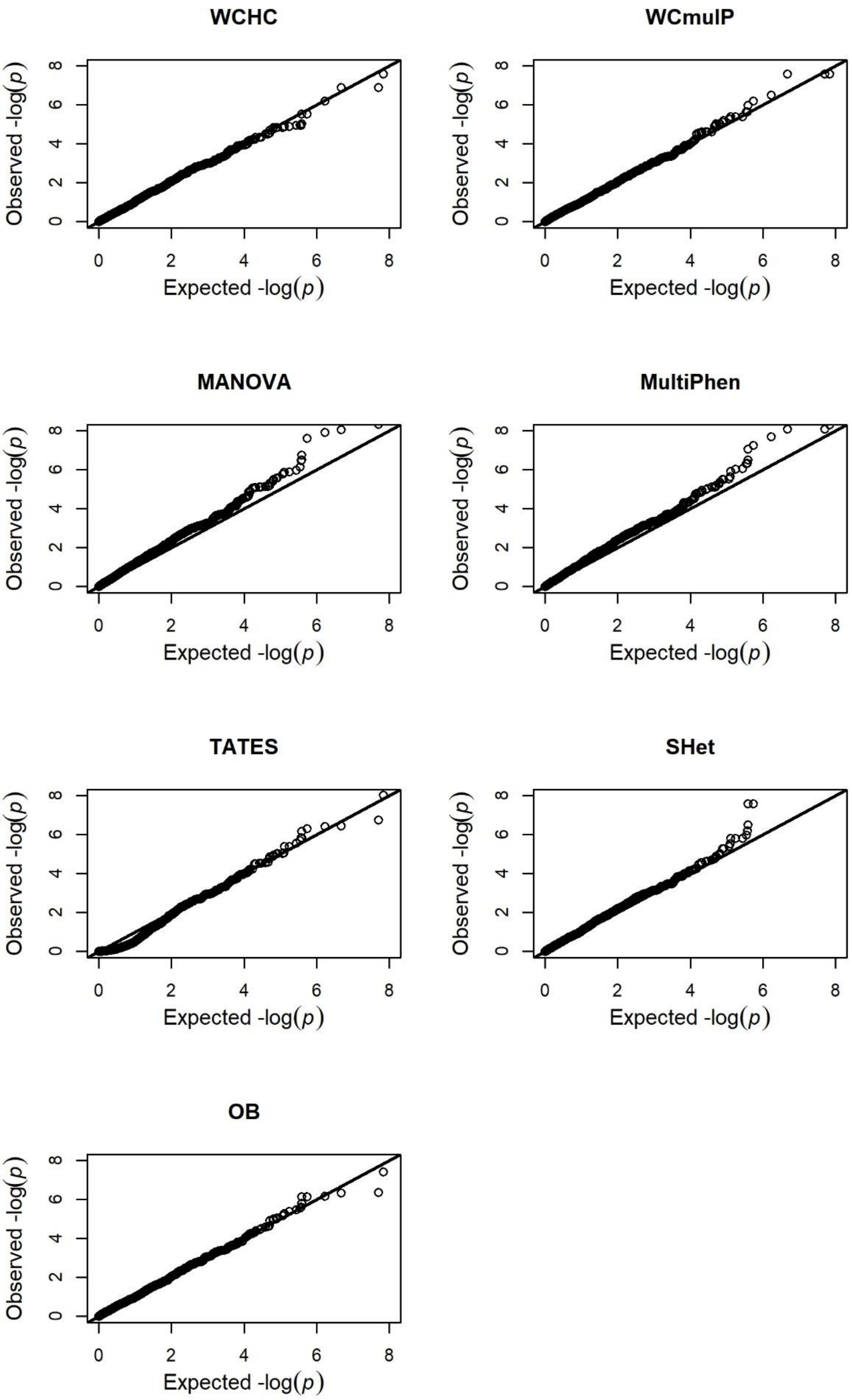

Supplementary Figure 4. Type I error rate comparisons of the seven methods in Q-Q plot. Sample size is $N = 1000$, the number of phenotypes is $M = 32$, the number of factors is $R = 1$, $c^2 = 0.5$, $\rho c^2 = 0.1$, and MAF = 0.3. The type I error rate of all the seven methods is estimated using 2000 replicated samples.

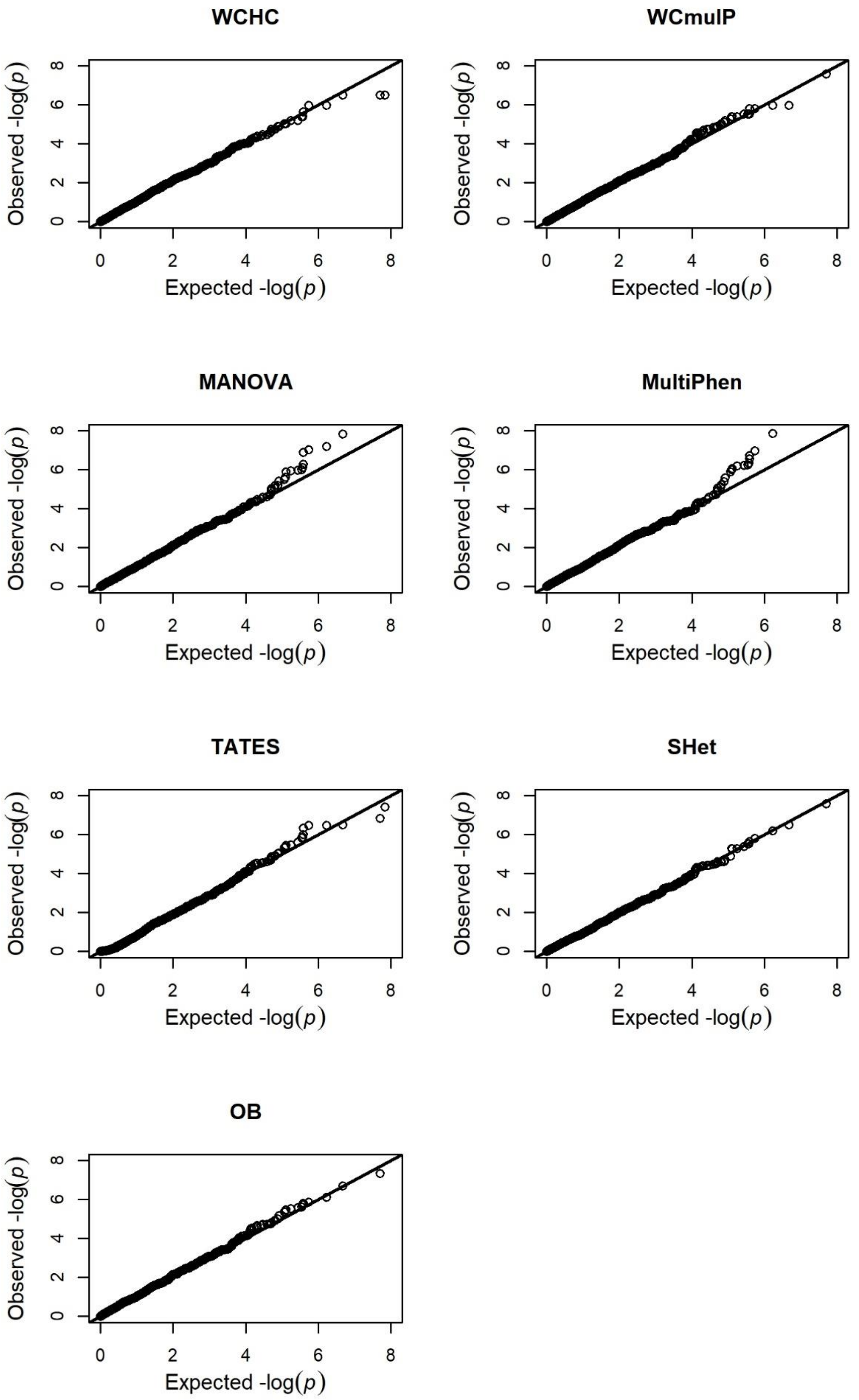

Supplementary Figure 5. Type I error rate comparisons of the seven methods in Q-Q plot. Sample size is $N = 1000$, the number of phenotypes is $M = 32$, the number of factors is $R = 2$, $c^2 = 0.5$, $\rho c^2 = 0.1$, and MAF = 0.3. The type I error rate of all the seven methods is estimated using 2000 replicated samples.

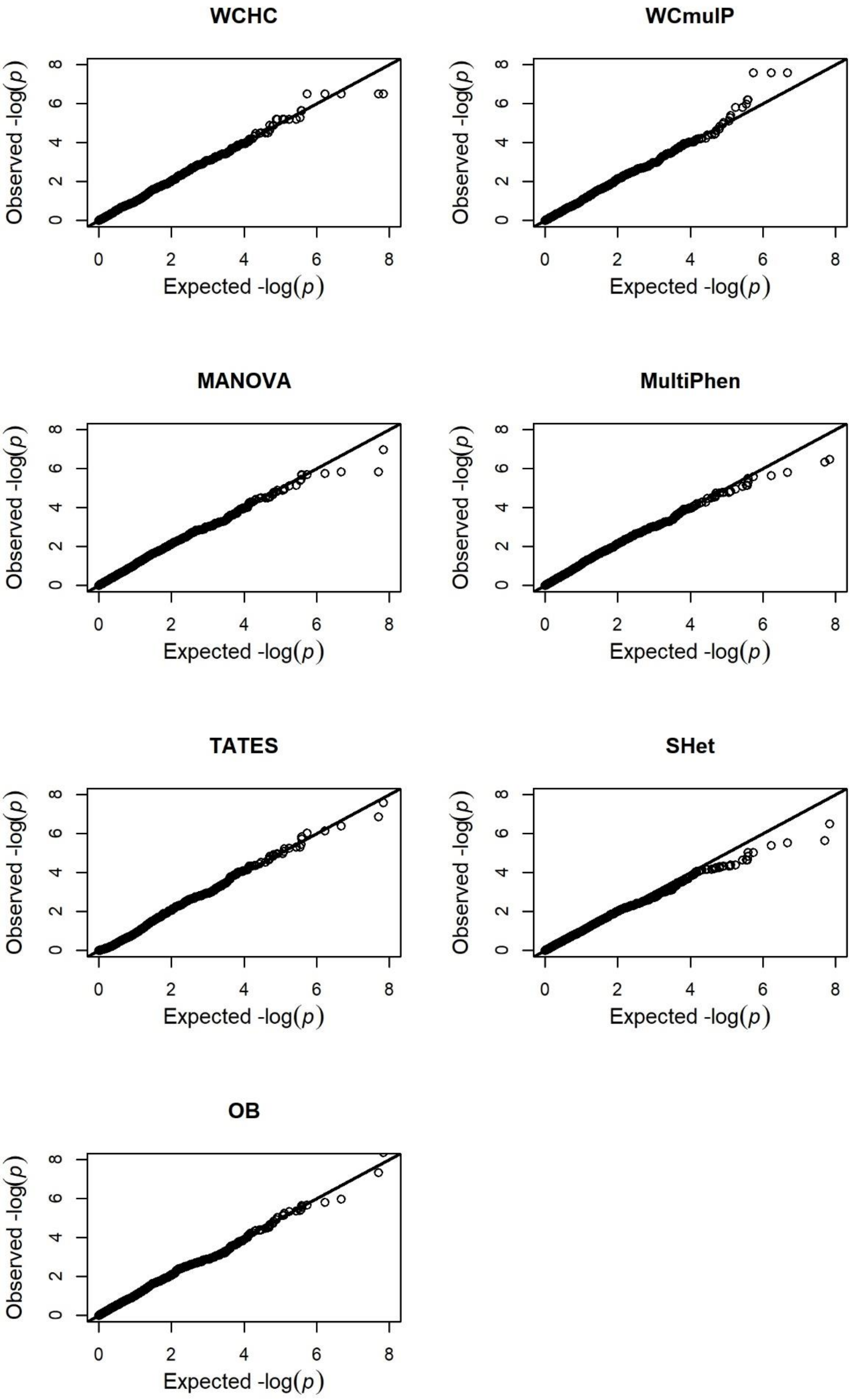

Supplementary Figure 6. Type I error rate comparisons of the seven methods in Q-Q plot. Sample size is $N = 1000$, the number of phenotypes is $M = 32$, the number of factors is $R = 4$, $c^2 = 0.5$, $\rho c^2 = 0.1$, and MAF = 0.3. The type I error rate of all the seven methods is estimated using 2000 replicated samples.

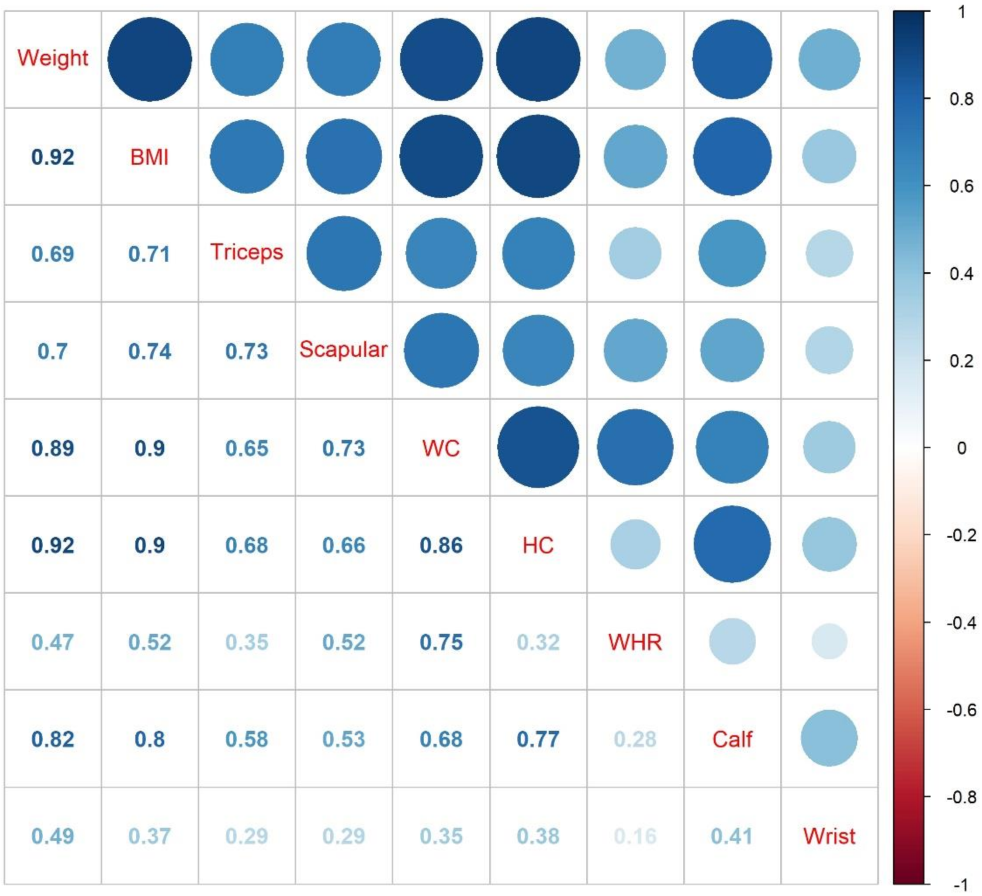

Supplementary Figure 7. Correlation coefficient matrix for obesity indicators in ARIC. BMI is body mass index; Triceps is average skinfold thickness of triceps brachii; Scapular is mean subscapular skinfold thickness; WC is waist; HC is hip girth; WHR is waist to hip ratio; Calf is calf girth; and Wrist is wrist breadth.
